# Development of an Extraction Method to Detect Hepatitis A Virus, Hepatitis E Virus, and Noroviruses in Fish Products

**DOI:** 10.3390/microorganisms11030624

**Published:** 2023-02-28

**Authors:** Catherine Hennechart-Collette, Océane Dehan, Audrey Fraisse, Sandra Martin-Latil, Sylvie Perelle

**Affiliations:** Laboratory for Food Safety, Université Paris-Est, ANSES, 14 rue Pierre et Marie Curie, CEDEX, F-94701 Maisons-Alfort, France

**Keywords:** fish products and fish meals, human norovirus, hepatitis A virus, hepatitis E virus, RT-qPCR, detection, process control

## Abstract

Viruses are a leading cause of foodborne disease worldwide. Hepatitis viruses (hepatitis A (HAV) and hepatitis E (HEV)) and human norovirus are recognized as the main viruses of public health concern in food hygiene. ISO 15216 approved procedures are not validated for detection of HAV and human norovirus in foodstuffs, such as fishes, leading to an inability to ensure the safety of these products. This study aimed to provide a rapid and sensitive method for detecting these targets in fish products. An existing method that includes proteinase K treatment was selected for further validation using artificially contaminated fish products, according to the recent international standard ISO 16140-4. Recovery efficiencies in pure RNA extracts of viruses ranged from 0.2% to 66.2% for HAV, 4.0% to 100.0% for HEV, 2.2% to 100.0% for norovirus GI, and 0.2% to 12.5% for norovirus GII. LOD_50_ values were between 144 and 8.4 × 10^4^ genome copies/g for HAV and HEV, and 10^4^ and 2.0 × 10^3^ copies/g for norovirus GI and norovirus GII, respectively. LOD_95_ values were between 3.2 × 10^3^ and 3.6 × 10^5^ genome copies/g for HAV and HEV, and between 8.8 × 10^3^ and 4.4 × 10^4^ genome copies/g for norovirus GI and norovirus GII, respectively. The method developed here was successfully validated in various fish products and can be applied for routine diagnostic needs.

## 1. Introduction

Enteric viruses, such as hepatitis viruses (hepatitis A (HAV) and hepatitis E (HEV)) and human norovirus, in fish and shellfish are a health concern worldwide. A significant number of foodborne illnesses have been reported after ingestion of raw shellfish and fish products [1,2,3]. Virus contamination occurs via consumption of contaminated food, direct person-to-person contact, contact with contaminated environmental surfaces [4,5], and for hepatitis E virus, via direct contact with infected animals [6,7,8]. Foodborne outbreak investigations have also revealed that food contamination mainly occurs in restaurants and in catering services [3,9,10,11,12,13]. 

Bivalves mollusks such as oysters are known to bioaccumulate pathogens that are the major etiological agents of gastroenteritis and have been involved in foodborne disease outbreaks [2]. In France, in addition to high-risk food categories such as bivalve mollusks, fishes are responsible for 8% of foodborne illnesses and about 4% of foodborne disease outbreaks due to viral agents [3]. In the United States, norovirus was implicated in 4% of seafood-associated infections between 1998 and 2015 [1].

Prevalence studies have shown that canned and processed seafood samples, such as fish fillets, and fresh or frozen fish have tested positive for norovirus in Italy [14] and in semi-processed fishery products in Belgium [15]. Noroviruses were detected in fresh and lightly cooked fishes collected during food handler-associated foodborne outbreaks in Tokyo, Japan [16]. A frozen tuna sample and tuna salad served in sandwiches have tested positive for hepatitis A (https://www.fda.gov/safety/recalls-market-withdrawals-safety-alerts/fda-investigates-findings-hepatitis-linked-frozen-tuna-fda-announcement#recall-announcement (accessed on 9 February 2018)) [17].

ISO 15216 procedures [18,19] propose molecular methods for detecting HAV and norovirus in high-risk food categories, such as bivalves mollusks, soft fruit, leaf, stem and bulb vegetables, bottled water, and on food surfaces. These methods are not validated for the detection of the targeted viruses in other foodstuffs, such as fishes, nor for the detection of other viruses, such as HEV in foodstuffs, leading to an inability to ensure the safety of these products.

To monitor the efficiency of a virus extraction procedure and to validate analyses, the ISO 15216 method advises the use of a process control virus and external amplification controls (EACs) as external control RNA to assess the inhibition of PCR amplification [18,19]. The process control used should exhibit similar morphological and physiochemical properties and environmental persistence to the target viruses, thus providing comparable extraction efficiency. The murine norovirus (MNV-1) has already been used successfully as a process control for HAV, HEV, and norovirus detection in shellfish, composite foodstuffs, dairy products or meat [20,21,22,23], and as a surrogate of enteric viruses [24].

The ISO 16140 procedure establishes the general principle as well as the technical protocol for the validation of alternative methods in the field of microbiological analysis of food. The recent international standard ISO 16140-4:2018 (microbiology of the food chain, method validation, part 4: protocol for single-laboratory method validation) [25] describes experimental designs to take into account the effect of various factors and their interactions, and reflects the variation within a single laboratory under routine conditions. 

The aim of this study was (i) to select a method for the detection of MNV-1, used as a surrogate virus in artificially contaminated fish products; and (ii) to validate the selected method for detecting HAV, HEV, and norovirus in different fish samples according to the experimental guidelines of the ISO 15216 procedure and the recent international standard ISO 16140-4 to ensure the safety of these products. 

## 2. Materials and Methods

### 2.1. Viruses 

HAV, HEV, norovirus, and MNV-1 stocks were obtained and titrated as described previously [26]. The genomic titers of viruses were determined using an RT-qPCR standard curve obtained with the 10-fold diluted in vitro HAV, HEV, norovirus GI, norovirus GII, and MNV-1 RNA transcripts. The in vitro RNA transcripts were quantified by measuring absorbance at 260/280 nm with a NanoDrop™. Virus titers were 2.90 × 10^9^ genome copies/mL for HAV, 1.00 × 10^7^ genome copies/mL for HEV, 1.60 × 10^7^ for norovirus GI, 1.30 × 10^7^ genome copies/mL for norovirus GII, and 8.90 × 10^11^ genome copies/mL for MNV-1, respectively. 

### 2.2. Artificial Contamination of Fish Products 

All samples were purchased from a local market. The selection of samples described in Table 1 takes account of different food processing factors (freezing, vacuum packaging, or canning). 

The experimental design from ISO 16140-4:2018 was applied (Table 2), and fish samples were artificially contaminated at four contamination levels for four viruses (HAV, HEV, norovirus GI, and norovirus GII) as described previously [26]. Concentration levels for viruses were achieved by using different dilution levels of the inoculum to obtain four rounded inoculation levels ranging from 2.9 × 10^3^ to 2.9 × 10^6^ genome copies for HAV, 10^3^ to 10^6^ genome copies for HEV, 1.6 × 10^3^ to 1.6 × 10^6^ for norovirus GI, and 1.3 × 10^3^ to 1.3 × 10^6^ for norovirus GII. 

Each separate portion of sample was contaminated with one virus of the four viruses and was co-inoculated with 8.9 × 10^8^ genome copies of MNV-1 (process control virus), just before adding elution buffer. For each sample, one uninoculated food sample spiked with sterile water was used as a negative control during the entire sample processing and viral detection procedure.

### 2.3. Sample Processing for Recovery of Viruses 

**Figure 1:**
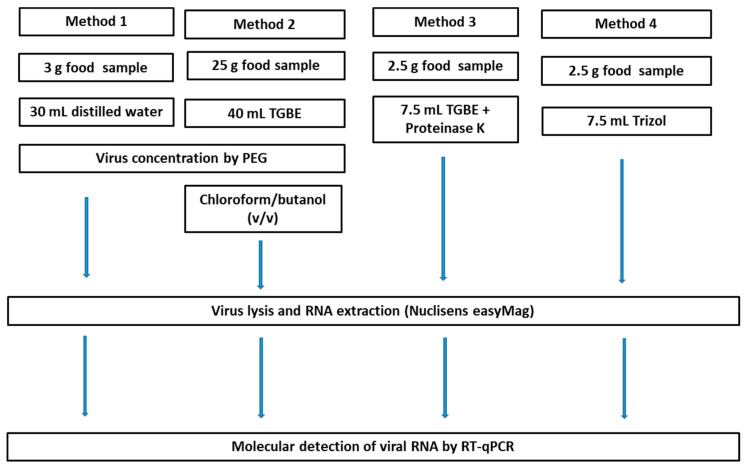
Flowchart of method 1 to method 4 evaluated for the detection of MNV-1 in raw salmon and smoked herring.

Four methods for recovering viruses from raw salmon and smoked herring were evaluated and performed three times. Figure 1 gives an overview of these four methods, and the details of the extraction methods are described below.

Method 1 was performed as previously described by Martin-Latil et al. [27] for the detection of viruses in foodstuffs of animal origin. Method 2 was adapted from the elution-concentration method described in the ISO 15216 procedure [18,19] for the detection of HAV and norovirus on soft fruits. Method 3 is a method adapted from standard ISO 15216 [18,19] for the detection of viruses in shellfish, and was implemented as previously described by Hennechart-Collette et al. [28] for norovirus detection in milk products. Method 4 is based on the use of TRIzol reagent, which has already been described for detecting enteric viruses in the dressed vegetables [29]. 

The RNA extracts were analyzed in duplicate with the RT-qPCR assays as described below. Uninoculated food samples were used as negative controls. 

### 2.4. Viral RNA Extraction and RT-qPCR Conditions

The viral RNA extraction, primers, and probe used as well as the one-step RT-qPCR amplifications assay have been described in [26].

A known amount of HAV, HEV, norovirus GI, or norovirus GII RNA transcript was used as an EAC to monitor RT-PCR inhibition in samples as described in [26,29,30].

### 2.5. Calculation and Interpretation of Results

HAV, HEV, norovirus GI, norovirus GII, and MNV-1 recovery rate percentages in spiked samples were calculated with reference to the corresponding standard curve. 

For methods 1 and 2, the MNV-1 recovery rates in spiked samples were calculated with reference to the corresponding standard curve and the following formula: quantity of virus recovered after spiking experiments/quantity of viral inoculum × 100.

For methods 3 and 4, virus recovery rates in spiked samples were calculated with reference to the corresponding standard curve and the following formula: quantity of virus recovered after spiking experiments × (volume of elution buffer)/quantity of viral inoculum × 100.

The comparison of the results obtained for EAC added to an aliquot of sample RNA with the results of EAC in the absence of sample RNA (i.e., in water) provides the percentage of RT-qPCR inhibition in each tested sample, as described in ISO 15216 procedures. HAV, HEV, norovirus GI, and norovirus GII inhibition rates in extracted RNA were calculated using the following formula: 100 − (quantity of external control RNA detected in sample/quantity of external control RNA detected in ultrapure water × 100).

### 2.6. Statistical Analysis 

Statistical analyses were performed using Statgraphics Centurion XVII version 17.1.04 software. Significance of results differences were determined with one-way analysis of variance (ANOVA, *p* value < 0.01) and a multiple comparison procedure (Fisher’s least-significant-differences (LSD)). The estimated probability of detection with 50% and 95% confidence (LOD_50_ and LOD_95_) was calculated by using the Wilrich POD LOD calculation software program (version 9, dated 23 September 2017) as in [26,30,31]. (www.wiwiss.fu-berlin.de/fachbereich/vwl/iso/ehemalige/wilrich/index.html (accessed on 23 September 2017)). 

## 3. Results

### 3.1. Comparison of Four Methods to Recover MNV-1 from Artificially Contaminated Fish Products

To develop a method for detecting enteric viruses in fish products, four methods were evaluated on raw salmon and smoked herring artificially contaminated by MNV-1 (which was used as a process control virus). The mean extraction yields obtained for MNV-1 by RT-qPCR are reported in Table 3.

The MNV-1 mean extraction yields calculated with pure RNA extracts ranged from 8.37% to 55.85% for method 1, from 3.03% to 36.77% for method 2, from 26.48% to 82.22% for method 3, and from 86.17% to 97.02% for method 4. 

Testing 10-fold diluted RNA extracts showed that extraction yields for MNV-1 in salmon improved by a factor of 1.34 using method 3, and in herring by a factor of 1.47 and 3.11 using methods 2 and 1, respectively. The dilution of RNA extracts did not enhance recovery rates of MNV-1 for salmon with methods 1, 2, and 4, nor for herring with methods 3 and 4.

To identify whether these four methods influenced the extraction yield of MNV-1, we compared the mean recovery rates of MNV-1 from artificially contaminated raw salmon and smoked herring samples. The method used did affect the extraction yield (one-way ANOVA; *p* = 0.000). A multiple comparison test showed that methods 3 and 4 had significantly higher extraction yields compared with methods 1 and 2. 

For each of these two methods (methods 3 and 4), the statistical analyses were performed to determine whether the dilution of RNA extracts enhanced recovery rates of MNV-1. Results showed that the average extraction efficiencies with pure RNA extracts compared to diluted RNA extracts for methods 3 and 4 were similar (one-way ANOVA; *p* = 0.8217 for method 3 and *p* = 0.8670 for method 4).

On the whole, methods 3 and 4 showed the highest average extraction efficiencies and no relevant amplification inhibition. Method 3 was preferred as the volume of eluate taken for RNA extraction is 10 times greater compared to method 4 and has the advantage of not using TRIzol reagent. 

### 3.2. Validation of the Selected Method for the Detection of HAV, HEV, and Norovirus in Samples

To characterize the selected method, the influence of experimental factors on the extraction yields of pathogenic viruses was assessed. 

#### 3.2.1. Mean Virus Recoveries of HAV, HEV, and Norovirus by Operator

**Figure 2:**
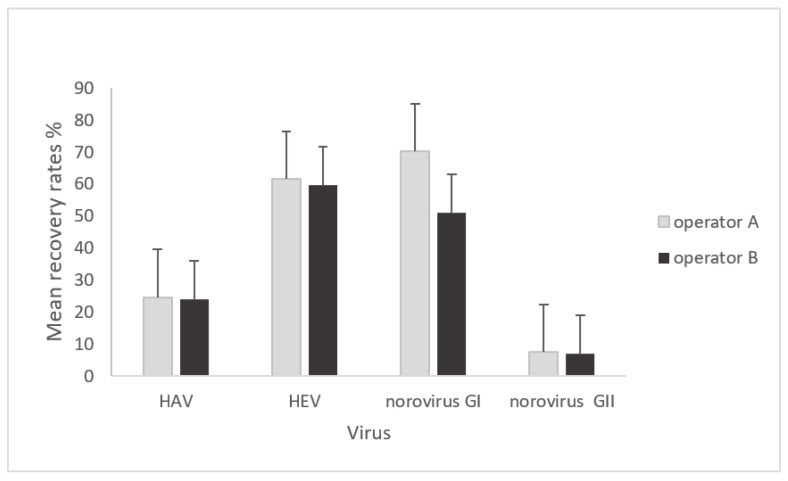
Comparison of mean recovery rates of HAV, HEV, norovirus GI, and norovirus GII according to the operator factor (operator A and operator B). For each virus, 8 analyses were performed by each operator (4 foodstuffs for all settings with all levels of inoculation) and pure RNA extracts were analyzed in duplicate with the RT-qPCR assay. Results are expressed as mean recovery rates (%) ± standard deviation (SD). one-way ANOVA; *p* = 0.0826.

The mean virus recoveries of HAV, HEV, norovirus, and MNV-1 obtained for all settings were compared for each operator (operator A and operator B), and statistical analyses were performed to identify whether the operator factor influenced the extraction recovery rates of viruses. Comparisons of mean recovery rates of HAV, HEV, norovirus GI, and norovirus GII according to the operator factor are reported in Figure 2.

For operator A, the means of recovery rates for all settings were 24.64%, 61.49%, 70.25%, and 7.67% for HAV, HEV, norovirus GI, and norovirus GII, respectively. For operator B, the means of recovery rates were 24.00%, 59.59%, 50.91%, and 6.95% for HAV, HEV, norovirus GI, and norovirus GII, respectively. 

The statistical analysis revealed that the operator factor did not influence virus recoveries from fish meal (one-way ANOVA; *p* = 0.0826). 

From now on, given this finding, the mean recoveries obtained for each virus are considered the mean recovery results obtained by the two operators (A and B).

#### 3.2.2. MNV-1 Recovery Rates and Recovery Rates of the EAC

According to the ISO 15216 procedure, the process control should be higher than 1%, and rates of inhibition in RNA extracted from food samples should be lower than 75%. 

MNV-1 was used as a process control to monitor the efficiency of the virus extraction method from fish products, and the data are presented in Table 4. MNV-1 was detected in 98% of RNA extracts analyzed (125 out of 128 extracts) and was recovered with an efficiency of between 14.76% (min) and 100% (max) for all allocated food settings and all targeted viruses. The process controls were less than 1% for the detection of HEV in the albacore tuna sample and for HAV detection in the tuna rillette samples. 

Implementation of the EAC corresponding to each viral target was used to examine RT-qPCR inhibition. Table 5 shows the inhibition rates of the RT-qPCR reaction for each sample, which varied from 12.93% to 84.50%. Higher rates of inhibition were observed for the tuna rillette sample.

#### 3.2.3. Viral Recovery Rates and Limits of Detection 

The recovery rates obtained for HAV, HEV, norovirus GI, and norovirus GII according to the inoculum level and repeated experiments (R1 to R4) are presented in Table 4. 

For all inoculum levels, the mean recovery rates with pure RNA extracts ranged from 0.18% to 66.19% for HAV, 4.04% to 100% for HEV, 2.18% to 100% for norovirus GI, and 0.17% to 12.49% for norovirus GII. As expected, for each sample, no viral RNA was detected in the uninoculated samples. The viral extraction yields from fish matrices were modified by a factor ranging from 0.1 to 8.9 by 10-fold dilution of RNA extracts (Table 4). Statistical analysis showed no differences between the average extraction efficiencies with pure RNA extracts and diluted RNA extracts (one-way ANOVA, *p* = 0.2296). 

The limit of detection (LOD) of the method was calculated for each virus and for each setting with the 12 samples. The LOD was estimated with pure RNA extracts. The LOD_50_ and LOD_95_ for HAV, HEV, norovirus GI, and norovirus GII are shown in Table 6.

The LOD_50_ for all settings was 144 genome copies/g for HAV, between 7.20 × 10^3^ and 8.40 × 10^4^ genome copies/g for HEV, 1.00 × 10^4^ genome copies/g for norovirus GI, and 2.00 × 10^3^ genome copies/g for norovirus GII.

The LOD_95_ for all settings was 6.40 × 10^3^ genome copies/g for HAV, between 3.20 × 10^3^ and 3.60 × 10^5^ genome copies/g for HEV, and between 8.80 × 10^3^ and 4.40 × 10^4^ genome copies/g for norovirus GI and norovirus GII.

### 3.3. Influence of Experimental Factors on Extraction Yield of Viruses

The statistical analysis revealed that the viral recovery rates varied according to the inoculated virus (one-way ANOVA; *p* < 0.001). More specifically, the multiple comparison tests showed that the recovery rates of HAV and norovirus GII were lower than the recovery rates obtained for norovirus GI and HEV. 

To determine whether the inoculation levels or the repeated experiments (R1 to R4) exhibited an impact on HAV, HEV, and norovirus recovery rates from food samples, statistical analyses were also performed according to the inoculation levels and repeated experiments used. Results of the one-way ANOVA showed that the recovery rates of viruses were not statistically different among the inoculation levels (one-way ANOVA; *p* = 0.5190 for HAV, *p* = 0.0712 for HEV, *p* = 0.1312 for norovirus GI, and *p* = 0.2955 for norovirus GII). However, the recovery rates of viruses were significantly different in the four repeated experiments (one-way ANOVA; *p* = 0.0002 for HAV, *p* < 0.001 for HEV, *p* = 0.0169 for norovirus GI, and *p* = 0.0015 for norovirus GII).

## 4. Discussion

Among the enteric viruses implicated in foodborne outbreaks, hepatitis viruses (HAV and HEV) and human norovirus represent a serious public health risk. A wide variety of foodstuffs has been implicated in viral outbreaks, including some high-risk foods such as shellfish. Water contaminated with fecal waste is the main transmission pathway of viruses in shellfish [32]. For fish immersed in contaminated water, it has been suggested that various organisms, such as viruses, penetrate into the organs and muscle [33] and could be responsible for virus transmission to humans. One study reported the presence of HEV RNA (genotype 3) in the sera and liver of bottlenose dolphins, which could result from environmental contamination of the dolphins’ food or from wastewater as a source of HEV exposure and infection [34].

Moreover, the most frequent route of virus transmission in foodborne outbreaks is secondary contamination via infected food handlers. Asymptomatic individuals shedding the virus play an important role in virus transmission, especially for raw and cooked products [13], and various fish products should be implicated in outbreak investigations. Fish types most often implicated in foodborne outbreaks were tuna [1], fish fillet, and raw salmon [16]. These high-risk matrices were selected in our study, with other foodstuffs that are consumed in a variety of forms and that are major components of almost all fish meal.

Few methods have been developed to recover viruses from fish products to date [14,35,36]. The general strategy for the detection of enteric viruses in food samples consists of three steps: virus extraction, purification of viral RNA, and molecular detection of the purified RNA. ISO 15216 offers validated methods for the detection and quantification of HAV and norovirus in seven food matrices, such as Pacific oysters (*Crassostrea gigas*) and common mussels (*Mytilus edulis*) [37]. However, to our knowledge, there are no data reporting the characterization of a method for detecting enteric viruses from fishes and fish meals. 

The experimental design from ISO 16140-4 was applied in this study for characterization of the method used to detect viruses in fish products. Another study including fresh or frozen seafood should be performed to assess the developed method. Studying the influence of the operator and of virus inoculation levels on virus recoveries improves the evaluation of method performance applied to fish samples. Analyzing a high number of samples, and taking into account different food processing factors and different matrix conditions (fat or presence of inhibitors), provides information on the variability of virus recoveries under different measurement conditions. In our study, 12 different fish product samples were tested. The number of samples tested is very important to characterize the method and to provide information on the variability of the results under different matrix measurements and to assess method performance. In this study, four methods for recovering viruses from fish products were evaluated, and a method based on proteinase K (method 3) was selected for validation. Method 4 presented the highest extraction efficiency for both salmon and smoked herring samples, while method 3 showed a low extraction efficiency for the salmon sample. The volume of eluate taken for RNA extraction with method 3 is 10 times greater compared to method 4. For all of these reasons, a routine method without TRIzol reagent was preferred. Method 3 with proteinase K treatment was selected for the detection of viruses from fish samples, but the discrimination between infectious and non-infectious viruses detected could not be evaluated. Methods using intercalating agent propidium monoazide (PMAxx™) pre-treatment with molecular assay allowed for assessing differences between infectious and non-infectious particles [38,39]. However, a PMAxx™-RT-qPCR method developed to selectively detect infectious murine norovirus particles in mussels showed that the approach is not sustainable with proteinase K treatment [39]. The proteinase K treatment coupled with heat treatment may damage the viral capsid [40,41,42]. 

The methods based on PEG precipitation (methods 1 and 2) showed mean recovery rates of MNV-1 ranging from 3.03% to 55.85% for pure RNA extracts. These data are consistent with those reported for PEG-based methods. MNV-1 recovered from figatelli and sausages ranged from 1.2% to 15.8% [27] and from 3.83% to 50.22% for multicomponent foods [26]. The proteinase K method selected had already been validated for virus detection in milk products [28,43]. The mean recovery of MNV-1 from milk products was between 51% and 81%, and between 5% and 100% for norovirus. Noroviruses and mengovirus were recovered at 3% and 1%, respectively, in shellfishes with a method based on proteinase K treatment [40,44]. This method is suitable for fish and milk products because they are both high-protein foods.

The LOD_95_ values of the proteinase K method used to detect viruses from fish products were between 10^3^ and 10^5^ genome copies/g for HAV and HEV, and between 10^3^ and 10^4^ genome copies/g for norovirus GI and norovirus GII. These LOD values are higher than those obtained for oyster, lettuce, or raspberries [37,45]. However, the LOD values for norovirus GI and norovirus GII in milk products were 10^5^ genome copies and 10^3^ genome copies/g, respectively [28], and are consistent with our results. 

In this study, the viral recovery rates varied depending on the inoculated virus and in the four repeated experiments. The origin of the inoculum, the quality of the fecal samples used, and the viral genotype may be important in explaining our virus recovery rates. Our result could also be explained by the study’s experimental design with the use of different fish food samples with different inoculation levels. The fish matrices might explain to some extent the differences in results between repeats. 

Use of various controls, such as the virus process control (MNV-1) and the EAC, which are described in the ISO 15216 procedure, was necessary to validate the results. MNV-1 was detected in 98% of RNA extracts and was successfully tested as a process control virus for detecting HAV, HEV, and norovirus in fish products. According to the recommendations of ISO 15216, the rates of RT-qPCR inhibition calculated with RNA extracted from food samples should be lower than 75%. In our study, RT-qPCR inhibition was higher than 75% with only one fish sample (tuna rillette). It is well known that the range of inhibition is the result of the different composition of food products, which can affect virus extraction [46,47,48,49,50].

To conclude, the method described herein can be used in addition to the method described in ISO 15216 for the detection of viruses in food matrices and can be applied for the detection of enteric viruses in fish products for routine diagnostic needs.

## Figures and Tables

**Table 1:** Selected food samples according to matrix type and processing factor.

Processing Factor	Matrix Types
Tin can	Fish dumpling Tuna Tuna rillette Salmon
Ready to eat meal	Salmon sorrel sauce
Raw and smoked (+4 °C)	Tuna albacore Cod Halibut Kipper Haddock
Fresh (+4 °C)	Shelled pink shrimp Surimi

**Table 2:** Experimental design for detection of enteric viruses (HAV, HEV, and noroviruses) in fish products adapted from the ISO 16140-4 procedure.

		Repeat Experiments			
Virus contamination levels		R1	R2	R3	R4
	High	Fish dumpling	Tuna	Tuna rillette	Kipper
	Medium	Tuna albacore	Shelled pink shrimp	Surimi	Haddock
	Low	Cod	Halibut	Salmon sorrel sauce	Salmon
	Very low	Shelled pink shrimp	Surimi	Haddock	Tuna albacore

**Table 3:** Comparison of mean recovery rates of MNV-1 from artificially contaminated salmon and herring samples processed using four study methods.

		Salmon		Herring	
Method	RNA Extracts	MNV-1 Extraction Yield (% ± SD)	Factor (F) (Diluted/Undiluted)	MNV-1 Extraction Yield (% ± SD)	Factor (F) (Diluted/Undiluted)
Method 1	Undiluted	55.85 ± 19.09	1.04	8.37 ± 7.18	3.11
	10-fold diluted	58.17 ± 24.93		26.10-± 13.51	
Method 2	Undiluted	3.03 ± 2.09	0.88	36.77 ± 28.16	1.47
	10-fold diluted	2.68 ± 1.29		54.28 ± 28.49	
Method 3	Undiluted	26.48 ± 13.46	1.34	82.22 ± 27.71	0.97
	10-fold diluted	35.57 ± 10.29		79.45 ± 27.93	
Method 4	Undiluted	97.02 ± 7.30	0.94	86.17 ± 21.43	1.05
	10-fold diluted	91.62 ± 13.00		93.50 ± 16.88	

Results are expressed as means of MNV-1 extraction yields (%) ± standard deviations (SD). The ratio (F) between the mean of extraction yields obtained with pure RNA extracts and those obtained with 10-fold diluted RNA extracts was calculated to determine whether the dilution of RNA extracts enhances mean extraction yields.

**Table 4:** Mean percent recovery calculated for four inoculum levels of HAV, HEV, and noroviruses in the presence of MNV-1.

Virus	Number of Genome Copies/Sample	RNA Extracts	Repeated Experiment R1 (% ± SD)	(F)	Repeated Experiment R2 (% ± SD)	(F)	Repeated Experiment R3 (% ± SD)	(F)	Repeated Experiment R4 (% ± SD)	(F)
HAV	2.9 × 10^6^	pure	32.06 ± 11.10 (4/4)	1.1	45.39 ± 10.06 (4/4)	1.1	0.18 ± 0.01 (2/4)	4.2	36.85 ± 5.50 (4/4)	1.34
		10-fold diluted	36.56 ± 10.86 (4/4)		49.48 ± 6.65 (4/4)		0.75 ± 0.62 (4/4)		49.36 ± 4.82 (4/4)	
	2.9 × 10^5^	pure	3.93 ± 0.63 (4/4)	1.7	23.89 ± 6.16 (4/4)	1.2	66.19 ± 6.38 (4/4)	>1.5	15.17 ± 3.14 (4/4)	3.08
		10-fold diluted	6.52 ± 1.49 (4/4)		28.73 ± 10.78 (4/4)		100.00 (4/4)		46.86 ± 36.07 (4/4)	
	2.9 × 10^4^	pure	12.13 ± 9.04 (4/4)	0.7	2.25 ± 2.55 (2/4)	5.4	40.52 ± 9.17 (4/4)	2.2	6.34 ± 3.34 (4/4)	1.1
		10-fold diluted	8.16 ± 6.75 (3/4)		12.09 ± 12.52 (2/4)		88.06 ± 61.91 (4/4)		7.03 ± 1.2 (2/4)	
	2.9 × 10^3^	pure	6.06 ± 5.21 (3/4)	0.1	21.13 ± 27.82 (4/4)	-	24.73 ± 25.49 (4/4)	4.0	24.51 ± 22.96 (4/4)	4.1
		10-fold diluted	0.40 (1/4)		nd (0/4)		100.00 (1/4)		100.00 (1/4)	
MNV-1	MNV		77.58 ± 31.29		71.21 ± 32.47		68.07 ± 52.74		43.25 ± 22.23	
	Total samples with recovery rates > 1%				30/32					
HEV	1.0 × 10^6^	pure	100 (4/4)	1.0	55.48 ± 13.42 (4/4)	0.97	19.69 ± 4.83 (4/4)	0.5	94.59 ± 10.82 (4/4)	0.73
		10-fold diluted	100 (4/4)		54.09 ± 18.35 (4/4)		9.93 ± 7.05 (4/4)		69.40 ± 33.75 (4/4)	
	1.0 × 10^5^	pure	nd (0/4)	-	34.46 ± 24.41 (4/4)	-	100 (4/4)	-	100 (4/4)	0.48
		10-fold diluted	nd (0/4)		nd (0/4)		0.99 (1/4)		48.06 ± 41.98 (3/4)	
	1.0 × 10^4^	pure	100 (1/4)	-	nd (0/4)	-	4.04 ± 6.21 (4/4)	-	87.15 (1/4)	-
		10-fold diluted	nd (0/4)		10.08 ± 0.55 (2/4)		nd (0/4)		nd (0/4)	
	1.0 × 10^3^	pure	nd (0/4)	-	nd (0/4)	-	4.39 (1/4)	-	nd (0/4)	-
		10-fold diluted	nd (0/4)		nd (0/4)		nd (0/4)		nd (0/4)	
MNV-1	MNV		61.18 ± 36.35		58.04 ± 24.79		45.40 ± 38.41		48.29 ± 27.67	
	Total samples with recovery rates > 1%				31/32					
norovirus GI	1.6 × 10^6^	pure	82.48 ± 13.79 (2/4)	1.21	97.30 ± 3.83 (4/4)	1.0	4.79 ± 3.07 (4/4)	8.9	63.01 ± 21.77 (4/4)	1.4
		10-fold diluted	100.00 (2/4)		100.00 (4/4)		42.51 ± 31.69 (4/4)		88.58 ± 13.24 (4/4)	
	1.6 × 10^5^	pure	49.27 ± 1.17 (2/4)	1.06	71.87± 32.51 (4/4)	0.93	68.60 ± 61.29 (4/4)	1.1	100.00 (4/4)	0.8
		10-fold diluted	52.47 ± 62.22 (2/4)		66.92 ± 54.90 (3/4)		77.11 ± 14.90 (4/4)		83.50 ± 32.93 (4/4)	
	1.6 × 10^4^	pure	2.18 (1/4)	-	nd (0/4)	-	97.32 (1/4)	-	34.41 ± 27.98 (3/4)	-
		10-fold diluted	nd (0/4)		nd (0/4)		nd (0/4)		nd (0/4)	
	1.6 × 10^3^	pure	nd (0/4)	-	nd (0/4)	-	nd (0/4)	-	nd (0/4)	-
		10-fold diluted	nd (0/4)		nd (0/4)		nd (0/4)		nd (0/4)	
MNV-1	MNV		100		100		100		80.29 ± 56.88	
	Total samples with recovery rates > 1%				32/32					
norovirus GII	1.3 × 10^6^	pure	11.34 ± 2.43 (4/4)	2.3	5.77 ± 1.30 (4/4)	1.0	0.17 ± 0.06 (4/4)	1.2	12.49 ± 1.21 (4/4)	1.1
		10-fold diluted	26.14 ± 33.00 (4/4)		5.67 ± 1.36 (4/4)		1.96 ± 0.37 (4/4)		14.09 ± 1.60 (4/4)	
	1.3 × 10^5^	pure	8.00 ± 1.23 (4/4)	1.0	6.51 ± 1.88 (4/4)	1.0	4.65 ± 0.51 (4/4)	0.7	7.66 ± 2.76 (4/4)	1.2
		10-fold diluted	8.45 ± 2.78 (4/4)		6.55 ± 3.68 (4/4)		3.31 ± 1.23 (3/4)		8.98 ± 5.29 (4/4)	
	1.3 × 10^4^	pure	9.09 ± 5.35 (4/4)	1.9	5.11 ± 5.22 (3/4)	2.1	10.43 ± 4.05 (4/4)	0.4	8.32 ± 2.04 (2/4)	-
		10-fold diluted	17.38 ± 20.49 (3/4)		10.77 ± 8.29 (2/4)		4.21 ± 5.91 (2/4)		nd (0/4)	
	1.3 × 10^3^	pure	6.92 ± 4.12 (2/4)	5.0	3.96 ± 0.33 (2/4)	-	nd (0/4)	-	nd (0/4)	-
		10-fold diluted	34.41 ± 19.47 (2/4)		nd (0/4)		nd (0/4)		nd (0/4)	
MNV-1	MNV		55.73 ± 8.56		51.00 ± 14.69		14.76 ± 11.79		46.42 ± 30.50	
	Total samples with recovery rates > 1%				32/32					

The mean percent recovery of operator A and B replicates was used for each inoculation level sample. RNA extracts were tested twice for each operator. nd: not detected. The ratios between the means of extraction yields obtained with undiluted RNA extracts and those obtained with 10-fold diluted RNA extracts were calculated to determine whether the dilution of RNA extracts enhanced the mean extraction yields (F). The mean percent recovery of viruses > 100% was reduced to 100%.

**Table 5:** Mean inhibition rates for HAV, HEV, and norovirus according to fish samples analyzed.

Sample Analyzed	Mean Inhibition Recoveries (% ± SD)
Fish dumpling	26.46 ± 33.77 (*N* = 8)
Albacore tuna	39.03 ± 32.27(*N* = 16)
Cod	26.60 ± 33.84 (*N* = 8)
Tuna	30.10 ± 30.26 (*N* = 8)
Shelled pink shrimp	36.83 ± 32.58 (*N* = 16)
Halibut	12.93 ± 16.12 (*N* = 8)
Tuna rillette	84.50 ± 34.35 (*N* = 8)
Surimi	25.82 ± 29.19 (*N* = 16)
Salmon sorrel sauce	20.56 ± 33.94 (*N* = 8)
Kipper	15.40 ± 34.47 (*N* = 8)
Haddock	15.73 ± 25.58 (*N* = 16)
Salmon	20.12 ± 19.57 (*N* = 8)

**Table 6:** Limit of detection (LOD_50_ and LOD_95_) for HAV, HEV, and norovirus according to the setting.

Target Virus	Genome Copies/g	R1	R2	R3	R4	All Settings
HAV	LOD_50_	600	3.8 × 10^3^	<1.2 × 10^3^ *	<1.2 × 10^3^ *	144
	LOD_95_	2.5 × 10^3^	1.7 × 10^4^	<1.2 × 10^3^ *	<1.2 × 10^3^ *	6.4 × 10^3^
HEV	LOD_50_	8.4 × 10^4^	1.2 × 10^2^	7.2 × 10^2^	7.6 × 10^3^	Between 7.2 × 10^2^ and 8.4 × 10^4^ **
	LOD_95_	3.6 × 10^5^	5.2 × 10^4^	3.2 × 10^3^	3.3 × 10^4^	Between 3.2 × 10^3^ and 3.6 × 10^5^ **
norovirus GI	LOD_50_	7.2 × 10^3^	1.9 × 10^4^	1.2 × 10^4^	3.5 × 10^3^	1 × 10^4^
	LOD_95_	3.1 × 10^4^	8.4 × 10^4^	5.2 × 10^4^	1.2 × 10^4^	4.4 × 10^4^
norovirus GII	LOD_50_	520	2.2 × 10^3^	1.5 × 10^3^	5.6 × 10^3^	2.0 × 10^3^
	LOD_95_	2.2 × 10^3^	9.6 × 10^3^	6.4 × 10^3^	2.5 × 10^4^	8.8 × 10^3^

* LOD_50_ and LOD_95_ were estimated. It was not possible to determine values because the LOD was not reached. ** LOD_50_ and LOD_95_ were estimated. It was not possible to calculate the LOD for all settings because determination of the zero failed during POD LOD calculation.

## Data Availability

The datasets generated during the current study are available from the corresponding author on reasonable request.
